# An Immunomics Approach to Schistosome Antigen Discovery: Antibody Signatures of Naturally Resistant and Chronically Infected Individuals from Endemic Areas

**DOI:** 10.1371/journal.ppat.1004033

**Published:** 2014-03-27

**Authors:** Soraya Gaze, Patrick Driguez, Mark S. Pearson, Tiago Mendes, Denise L. Doolan, Angela Trieu, Donald P. McManus, Geoffrey N. Gobert, Maria Victoria Periago, Rodrigo Correa Oliveira, Fernanda C. Cardoso, Guilherme Oliveira, Rie Nakajima, Al Jasinskas, Chris Hung, Li Liang, Jozelyn Pablo, Jeffrey M. Bethony, Philip L. Felgner, Alex Loukas

**Affiliations:** 1 Centre for Biodiscovery and Molecular Development of Therapeutics, Australian Institute of Tropical Health and Medicine, Queensland Tropical Health Alliance Laboratory, James Cook University, Cairns, Queensland, Australia; 2 Instituto Nacional de Ciência e Tecnologia em Doenças Tropicais, Centro de Pesquisas René Rachou, Instituto Fiocruz, Belo Horizonte, Minas Gerais, Brazil; 3 QIMR Berghofer Medical Research Institute, Brisbane, Queensland, Australia; 4 Federal University of Minas Gerais, Belo Horizonte, Minas Gerais, Brazil; 5 Institute for Molecular Biosciences, The University of Queensland, St Lucia, Queensland, Australia; 6 University of California Irvine, Irvine, California, United States of America; 7 George Washington University, Washington, D.C., United States of America; Uniformed Services University, United States of America

## Abstract

Schistosomiasis is a neglected tropical disease that is responsible for almost 300,000 deaths annually. Mass drug administration (MDA) is used worldwide for the control of schistosomiasis, but chemotherapy fails to prevent reinfection with schistosomes, so MDA alone is not sufficient to eliminate the disease, and a prophylactic vaccine is required. Herein, we take advantage of recent advances in systems biology and longitudinal studies in schistosomiasis endemic areas in Brazil to pilot an immunomics approach to the discovery of schistosomiasis vaccine antigens. We selected mostly surface-derived proteins, produced them using an *in vitro* rapid translation system and then printed them to generate the first protein microarray for a multi-cellular pathogen. Using well-established Brazilian cohorts of putatively resistant (PR) and chronically infected (CI) individuals stratified by the intensity of their *S. mansoni* infection, we probed arrays for IgG subclass and IgE responses to these antigens to detect antibody signatures that were reflective of protective vs. non-protective immune responses. Moreover, probing for IgE responses allowed us to identify antigens that might induce potentially deleterious hypersensitivity responses if used as subunit vaccines in endemic populations. Using multi-dimensional cluster analysis we showed that PR individuals mounted a distinct and robust IgG1 response to a small set of newly discovered and well-characterized surface (tegument) antigens in contrast to CI individuals who mounted strong IgE and IgG4 responses to many antigens. Herein, we show the utility of a vaccinomics approach that profiles antibody responses of resistant individuals in a high-throughput multiplex approach for the identification of several potentially protective and safe schistosomiasis vaccine antigens.

## Author Summary

Schistosomiasis is a neglected tropical disease that kills as many as 300,000 people each year. Mass drug administration is widely used to control schistosomiasis, but fails to prevent rapid reinfection in endemic areas. There is a desperate need for a prophylactic vaccine; however, very few candidates have been developed. Herein, we take advantage of recent advances in systems biology and longitudinal studies in schistosomiasis endemic areas to pilot an immunomics approach to the discovery of vaccine antigens. The emerging field of immunomics enables the determination of an “antibody signature” to a pathogen proteome for both resistant and susceptible individuals. We constructed the first protein microarray for a multi-cellular pathogen and probed it with sera from naturally resistant vs. susceptible individuals from a high transmission area in Northeastern Brazil. Using multi-dimensional cluster analysis, we showed that resistant individuals mounted a distinct and robust IgG1 antibody signature to a small set of newly discovered and well-characterized surface antigens in contrast to infected individuals. This antigen discovery strategy can lead to identification of several potentially protective and safe schistosomiasis vaccine antigens.

## Introduction

Schistosomiasis is a chronic, often debilitating, parasitic disease affecting over 200 million people worldwide and killing at least 300,000 people annually [1]. The disability adjusted life years (DALYs) lost to schistosomiasis are potentially as high as 70 million [2], [3]. Adult flukes live in the portal and mesenteric veins (*Schistosoma mansoni* and *S. japonicum*) or in the veins of the bladder (*S. haematobium*), as male/female pairs, and survive for many years producing hundreds of fertilized eggs per day. Severe morbidity results from the host immune responses to eggs that become trapped in the tissues, including periportal fibrosis, portal hypertension, urinary obstruction and bladder carcinoma [4].

Currently, chemotherapy with praziquantel (PZQ) is the standard treatment for schistosomiasis. Control programs based on mass drug administration (MDA) with PZQ have been complicated by rapid and frequent re-infection of treated individuals, and the difficulties and expense of maintaining continuous MDA over the long term [5]. Additionally, resistance to PZQ can be induced in the laboratory [6], and field isolates displaying reduced susceptibility to the drug have been reported (reviewed in [7]). Despite recent large-scale MDA efforts [8], integrated control programs aimed at limiting schistosomiasis by improving education and sanitation, molluscicide treatment programs to reduce the population of the intermediate snail host, and chemotherapy have had limited success [5], [9]. A vaccine that induces long-term immunity to schistosomiasis is therefore necessary to reach our goals of elimination.

The high prevalence of chronic schistosomiasis in endemic populations suggests that sterile immunity is rarely generated. However, the decline in infection intensity at an earlier age in populations with high infection intensity [10], and more rapid development of resistance to re-infection after several rounds of PZQ treatment (drug-induced resistance) [11], indicates that non-sterilizing immunity, though slow to develop, can occur. Despite the slow acquisition of non-sterile immunity over time, there is still an urgent need for a prophylactic vaccine, particularly one that targets children, who represent the most at-risk population.

There are two major obstacles to the development of an efficacious schistosomiasis vaccine. The first is the ability of schistosomes to employ a range of strategies for evasion of the host immune response. Central to the parasite's ability to evade immune clearance is its unique host-interactive outer surface, or tegument, consisting of a single, contiguous, double-bilayered membrane that covers the entire worm [12]. At this interface essential functional interactions with the human host occur, such as nutrient uptake and environmental sensing. The tegument is also the primary site where the parasite defends itself against immune recognition. The host-interactive surface is indeed the target of the few successful examples of metazoan parasite vaccines, such as those targeting the cattle tick *Boophilus microplus* [13], the gastrointestinal nematode *Haemonchus contortus* [14] and the cestodes, *Taenia ovis* [15] and *Echinococcus granulosus* [16]. The second major obstacle to the development of a schistosomiasis vaccine resides in the historic approach to antigen discovery for this pathogen. To date, only one schistosomiasis vaccine, rSh28GST from *S. haematobium*, is currently in phase I clinical trials, where it was shown to be safe and immunogenic [17]. Other vaccine antigens for *S. mansoni* are in pre-clinical and clinical development [18], [19], with safety and immunogenicity results yet to be reported. We [19]–[21] and others [22], [23] have advocated for the utility of tegument proteins as a basis for subunit vaccines against schistosomiasis. Three of the current lead candidate antigens are located in the tegument and are exposed on the surface of the parasite [24]–[26]. The genomes for the three major human schistosomes have been sequenced [27]–[29], and coupled with proteomic studies that characterised the surface proteomes of *S. mansoni* [30] and *S. japonicum* [31], have provided researchers with a catalogue of proteins for discovery and development of a new panel of vaccine antigens.

To best mine this extensive proteomic data and identify antigens that are preferentially recognised by antibodies from naturally resistant individuals resident in areas of high transmission for schistosomiasis, we have utilized a clinical cohort of individuals referred to as Putative Resistants (PRs). As part of a ten year longitudinal study of individuals from high *S. mansoni* transmission areas of Brazil, we identified a cohort of individuals who were constantly exposed to *S. mansoni* infection as determined by extensive water contact and epidemiological studies, but remained egg-negative over the course of the study [32]–[34]. In addition to this unique epidemiological profile, these individuals mounted an immune response that displayed a markedly different phenotype from that of chronically infected (CI) individuals [35]–[37]. Indeed, two of the current antigens in pre-clinical development - *Sm*-TSP-2 [26] and *Sm*29 [24] – were discovered as a result of their selective recognition by PR subjects, highlighting their utility as a tool for discovery of protective vaccine antigens.

Herein, we describe the screening of the first protein microarray for a human helminth parasite, and only the second such array for a eukaryotic parasite other than *Plasmodium sp*. We developed a targeted array consisting primarily of tegument derived proteins from both *S. mansoni* and *S. japonicum* [38]and screened the array with sera from PR and CI individuals with low, medium and high intensity infections, and then compared and contrasted antibody/antigen recognition profiles to determine antibody signatures that characterised natural resistance or susceptibility to infection. We assessed IgG subclass and IgE responses such that potential vaccine antigens could be assessed for their protective properties as well as their safety profiles in terms of exacerbating allergic IgE responses [39]. We showed that individuals with medium and heavy intensity infections generally recognized more antigens and with higher magnitude than did PR individuals and those with low infection intensities. Moreover, we found that PR individuals did not mount an intense IgE response to these antigens compared to CI individuals, but instead produced IgG1/3 (cytophilic) antibody responses to only a few membrane bound antigens. We successfully utilized this approach to identify new, and confirm existing, vaccine antigens via their selective IgG1/IgG3 recognition profiles by PR individuals in the absence of a potentially deleterious IgE response.

## Results

### Schistosome protein microarray production and quality control

Details of the microarray production and associated QC have been described elsewhere [38] and are shown in Figure S1 and Table S1, however this is the first report that describes probing of the microarray with human sera. We included on the array two dilutions of the ubiquitously immunoreactive Epstein Barr virus protein EBNA-1 at two concentrations, 0.1 and 0.3 mg/ml, as a non-schistosome control for the rapid translation system (RTS) used to express schistosome recombinant proteins. Both antigens were consistently recognized by IgG1 antibodies from all individuals tested (Figure S2), indicating that sera from all cohorts were of sufficient integrity for further analyses.

### Antibody profiles correlate with infection intensity

**Figure 1:**
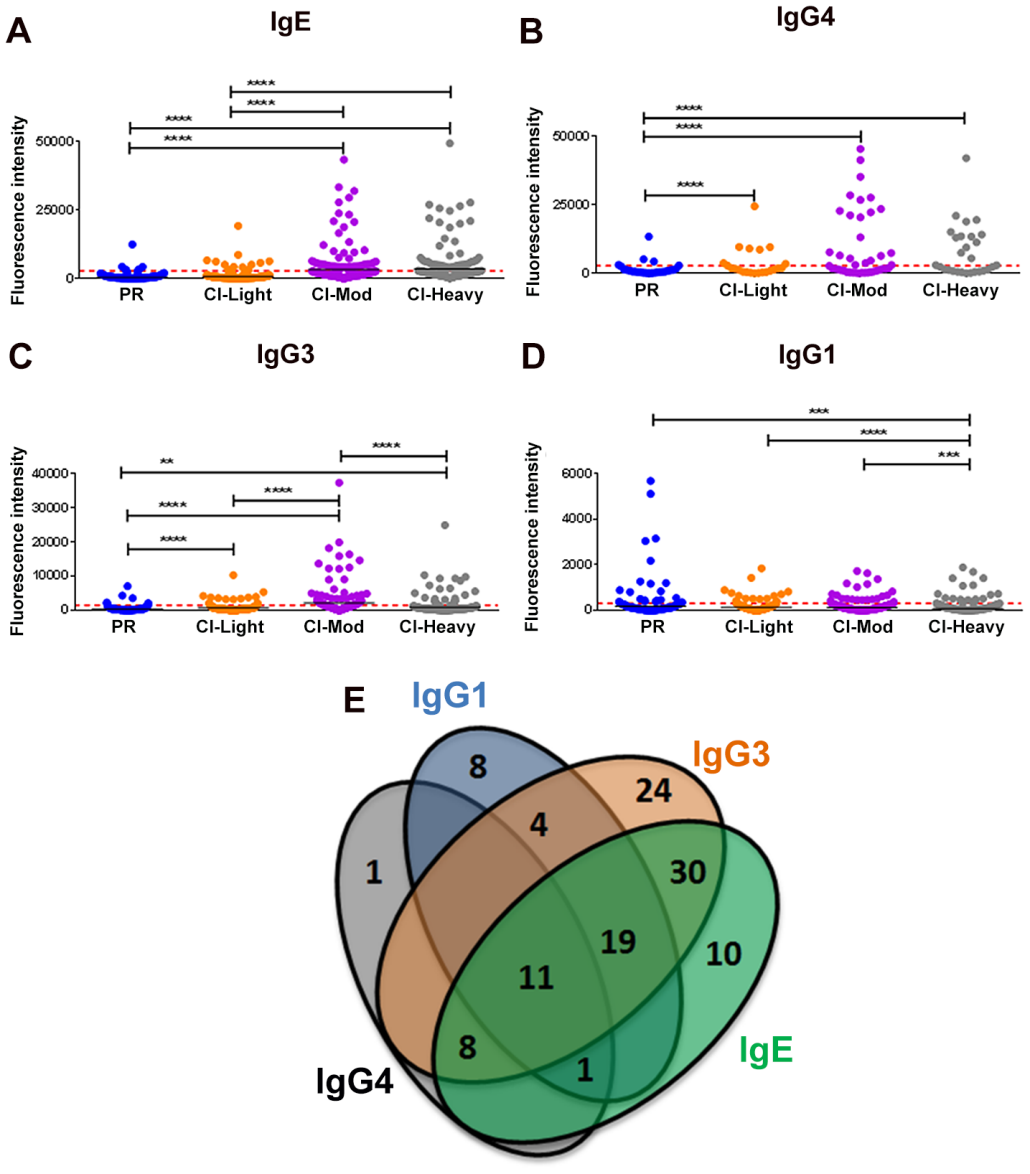
Isotype and subclass-specific antibody recognition profiles of PR and CI individuals to schistosome antigens printed on the proteome microarray. The average adjusted signal intensity for IgE (A), IgG4 (B), IgG3 (C) and IgG1 (D) antibody responses of individual protein microarray features Cohorts of individuals that are putatively resistant (PR) to *S. mansoni* infection, or have low (CI-Light), moderate (CI-Mod) and heavy (CI-Heavy) intensity infections (based on eggs per gram of feces) were graphed. Red lines denote the cut-offs for each antibody isotype/subclass calculated by determining the average of the signal intensity for control spots on the microarray that contained no DNA (No DNA controls). Statistical analysis was performed using one-way ANOVA with Dunn's multiple comparisons test. *p<0.05; **p<0.01, ***p<0.001, ****p<0.0001. Venn diagrams (E) representing the combined data of Figures 1A-D. Values in the diagram show the protein microarray features recognized by each isotype/subclass.

Given the distinct roles of different immunoglobulin isotypes and IgG subclasses in chronic helminth infections, and to gain a comprehensive picture of antibody reactivity from PR versus CI individuals, we analyzed IgG1, IgG3, IgG4 and IgE responses to soluble worm antigen preparation (SWAP) and a panel of schistosome antigens. PR subjects mounted the strongest anti-SWAP IgG1 and IgG3 responses whereas the moderate and heavily infected groups mounted the strongest IgG3 responses to SWAP (Figure S3). One hundred and sixteen (116) from a total of 215 (54%) RTS proteins spotted were recognized by at least one antibody isotype/subclass from at least one cohort of exposed individuals (reactive proteins) (Table S2 and Table S3). Individuals with medium and heavy intensity infections generally recognized more antigens and with stronger SI than did PR individuals and those with low infection intensities (Figures 1A-D). CI-Mod and CI-Heavy cohorts had significantly higher IgG4, IgG3 and IgE responses than PR and CI-Light cohorts (Figures 1A-C) (*p<0.05; **p<0.01, ***p<0.001, ****p<0.0001). In contrast, the PR cohort had significantly higher IgG1 (Figure 1D) responses than CI-Mod and CI-Heavy cohorts, although the total number of antigens above the cut-off was lower for this antibody subclass. In general, there was a strong correlation between infection intensity and the number of antigens recognized by combinations of IgG3, IgG4 and IgE from infected individuals (Table S4 and Table S5). Of the 116 reactive proteins (RTS and recombinants) 41 were recognized by just a single antibody isotype/subclass: 8 proteins were recognized by only IgG1, 24 proteins were recognized by only IgG3, one protein was recognized by only IgG4 and 10 proteins were recognized by only IgE. Eleven proteins were recognized by all antibody isotypes/subclasses (Figure 1E) (Table S2).

### IgE responses

**Figure 2:**
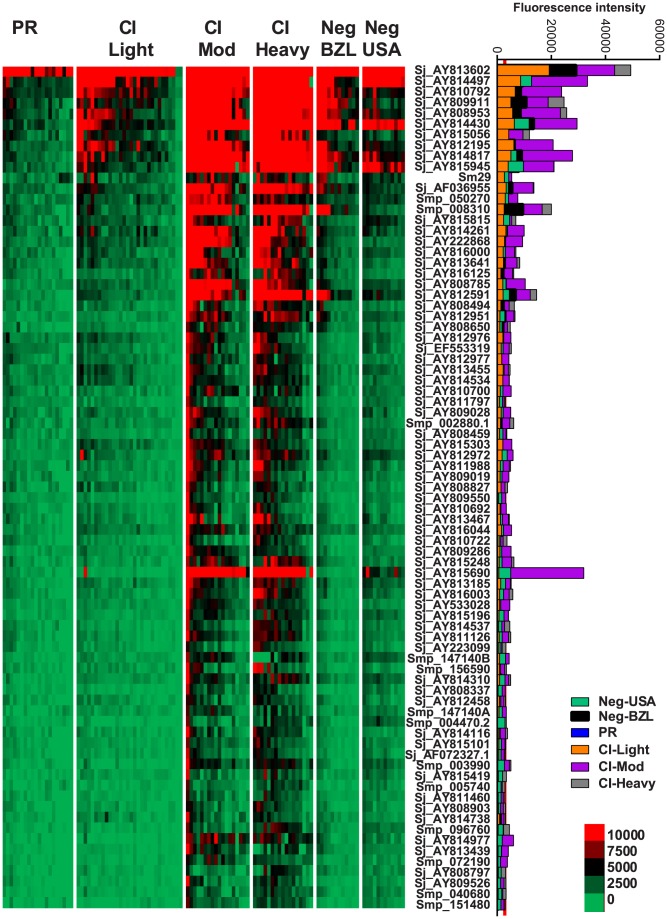
IgE reactivity profiles of resistant and susceptible human cohorts to *Schistosoma* proteins printed on a proteome microarray. Heatmap showing IgE responses of individual subjects (columns) in each cohort to 79 recombinant antigens (rows) printed on the microarray. Green represents no immunoreactivity through to red symbolizing strong immunoreactivity. The bar graph depicts the average signal intensity of each cohort. Proteins are ordered based on SI mean, highest to lowest, in the groups. Human cohorts represented: Putative Resistant (n = 20), CI-Light (n = 30), CI-Mod (n = 18) and CI-Heavy (n = 17) non-endemic Brazilian volunteers (n = 12), non-endemic North American volunteers (n = 12). The red line is the cut-off for reactivity, calculated as one standard deviation of the mean of the no-DNA control spots printed on the array and probed with anti-IgE. All of the proteins showed significant differences between at least two of the schistosome exposed groups (PR, CI-Light, CI-Mod and CI-Heavy) as calculated by Kruskal-Wallis test with Dunn's multiple comparison post-test (Table S4).

IgE responses were detected to 79 different antigens (Figure 2, Table S2) and most of these were restricted to the CI-Mod and CI-Heavy groups. Significant differences (P≤0.05) between mean antibody responses from 2 or more of the endemic groups were detected to all 79 proteins (Tables S2–4). The only purified recombinant protein (non-RTS) that was the target of an IgE response was *Sm*29. Antigens for which the strongest IgE responses were detected included proteins that were predicted and/or proven to be located on the tegument membrane (including tetraspanins, Ly6/CD59-like proteins such as *Sm*29, and glucose transporters) and predicted intracellular proteins including mitochondrial enzymes, chaperones and glycolytic enzymes such as triose phosphate isomerase (Table S1 and Table S2).

### IgG subclass responses

**Figure 3:**
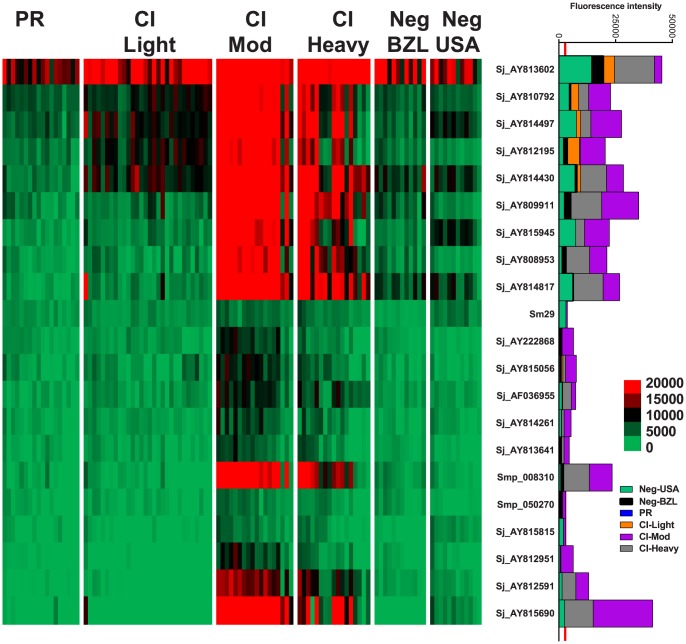
IgG4 reactivity profiles of resistant and susceptible human cohorts to *Schistosoma* proteins printed on a proteome microarray. Heatmap showing IgG4 responses of individual subjects (columns) in each cohort to 23 recombinant antigens (rows) printed on the microarray. Green represents no immunoreactivity through to red symbolizing strong immunoreactivity. The bar graph depicts the average signal intensity for each cohort. Proteins are ordered based on SI mean, highest to lowest, in the groups. Human cohorts represented: Putative Resistant (n = 20), CI-Light (n = 30), CI-Mod (n = 18) and CI-Heavy (n = 17) non-endemic Brazilian (n = 12) and non-endemic North Americans (n = 12). The red line is the cut-off for reactivity, calculated as one standard deviation of the mean of the no-DNA control spots printed on the array and probed with anti-IgG4. NS represents no significant differences between endemic groups calculated by Kruskall Wallis with Dunn's multiple comparison test. All other proteins showed significant differences between at least two of the schistosome exposed groups (PR, CI-Light, CI-Mod and CI-Heavy) (Table S4).

IgG4 responses were detected to 21 proteins (Figure 3) – 20 RTS proteins and purified recombinant *Sm*29 expressed in *E. coli*. Significantly different IgG4 responses were detected for all reactive antigens between at least two of the endemic cohorts (Table S3, Table S4). *Sm*29 was recognized weakly by IgG4 but was considered a cross-reactive protein because the US non-endemic control group had a low level IgG4 response against this protein (Figure 3, Table S2).

**Figure 4:**
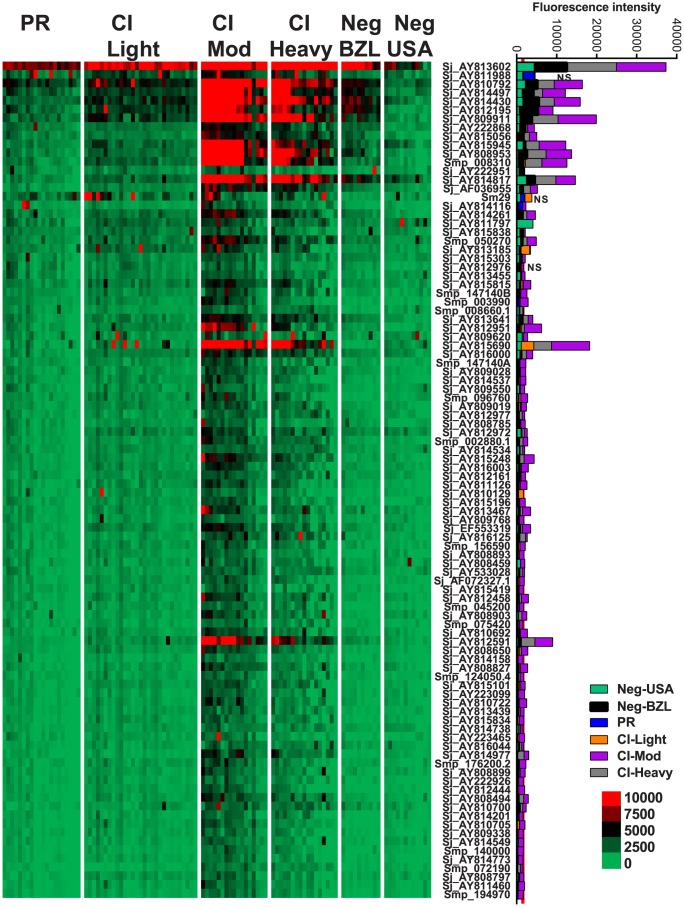
IgG3 reactivity profiles of resistant and susceptible human cohorts to *Schistosoma* proteins printed on a proteome microarray. Heatmap showing IgG3 responses of individual subjects (columns) in each cohort to 98 recombinant antigens (rows) printed on the microarray. Green represents no immunoreactivity through to red symbolizing strong immunoreactivity. The bar graph depicts the average signal intensity with mean standard deviation of each cohort. Proteins are ordered based on SI mean, highest to lowest, in the groups. Human cohorts represented: Putative Resistant (n = 20), CI-Light (n = 29), CI-Mod (n = 17) and CI-Heavy (n = 17). non-endemic Brazilian (n = 10), non-endemic North Americans (n = 12). The red line is the cut-off for reactivity, calculated as one standard deviation of the mean of the no-DNA control spots printed on the array and probed with anti-IgG3. NS represents no significant differences between endemic groups calculated by Kruskall Wallis with Dunn's multiple comparison test. All other proteins showed significant differences between at least two of the schistosome exposed groups (PR, CI-Light, CI-Mod and CI-Heavy) (Table S4).

IgG3 responses were detected to 96 proteins, 95 of which were RTS and 1 *E. coli*-derived purified recombinant proteins (Figure 4, Table S2). Of the 96 reactive proteins, only 3 displayed no significant differences between the cohorts (Figure 4, Table S4).

**Figure 5:**
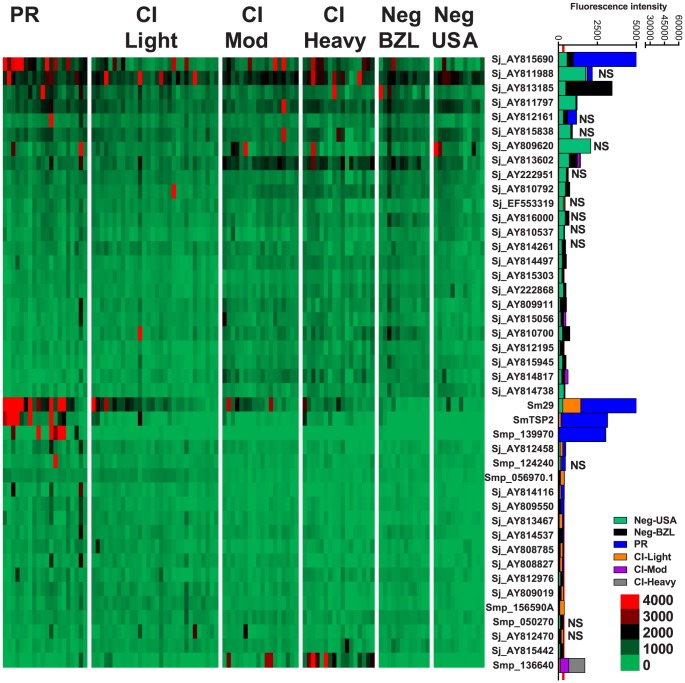
IgG1 reactivity profiles of resistant and susceptible human cohorts to *Schistosoma* proteins printed on a proteome microarray. Heatmap showing IgG1 responses of individual subjects (columns) in each cohort to 45 recombinant antigens (rows) printed on the microarray. Green represents no immunoreactivity through to red symbolizing strong immunoreactivity. The bar graph depicts the average signal intensity with mean standard deviation of each cohort. Proteins are ordered based on SI mean, highest to lowest, in the groups. Human cohorts represented: non-endemic Brazilian (n = 12), Putative Resistant (n = 20), CI-Light (n = 30), CI-Mod (n = 18) and CI-Heavy (n = 17). The red line is the cut-off for reactivity, calculated as one standard deviation of the mean of the no-DNA control spots printed on the array and probed with anti-IgG1. NS represents no significant differences between endemic groups calculated by Kruskall Wallis with Dunn's multiple comparison test. All other proteins showed significant differences between at least two of the exposed groups (PR, CI-Light, CI-Mod and CI-Heavy) (Table S4).

IgG1 responses were detected to 43 proteins (Figure 5, Table S2), including purified recombinant *Sm*29 and *Sm*-TSP-2. Significantly different IgG1 responses between endemic cohorts were detected for 31 of these reactive antigens (Table S4). Twenty-two proteins were the targets of an IgG1 response in the PR cohort that was significantly different to at least one of the CI groups (Table S4). The most robust of these PR-specific IgG1 responses were aimed at the two positive control recombinant proteins, *Sm*-TSP-2 and *Sm*29, and the RTS protein Smp_139970, a calmodulin-3 like protein that we have termed *Sm*-CAM-3. *Sm*-CAM-3 shared 46% and 23% amino acid identities with its closest *S. mansoni* and primate (macaque) homologues respectively (Figure S4).

Correlations between different isotype responses to the same proteins were calculated (Table S5). The strongest correlations detected (*r^2^*>0.9) were between IgG4/IgE responses in all the schistosome-exposed cohorts (P<0.0001, Figure S5) and IgG3/IgG4 and IgG3/IgE responses in the CI-Mod and CI-Heavy cohorts.

### Cluster analysis

**Figure 6:**
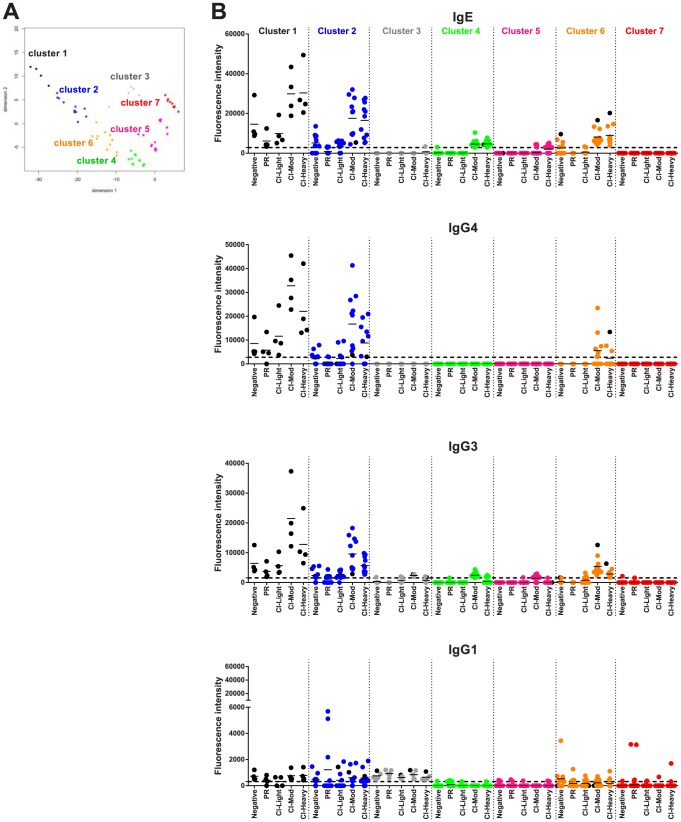
Immunoreactivity of *Schistosoma* proteins by multi-dimensional cluster analysis. (A) Multi-dimensional clustered distribution of all proteins according to the antibody isotype/subclass responses in distinct cohorts. To identify clusters containing proteins with the same antibody reactivity profiles, a distance matrix estimated from the pairwise Euclidian distance of log transformed signal intensity (SI) was generated for each antigen based on the cut-off values for each antibody isotype/subclass in the different cohorts. Proteins formed 7 clusters, defined by the following colors: cluster 1 - black (4 proteins); cluster 2 – blue (11 proteins); cluster 3 – grey (5 proteins); cluster 4 – green (31 proteins); cluster 5 – magenta (47 proteins); cluster 6 – orange (11 proteins) and cluster 7 – red (106 proteins). (B) Two-dimensional depiction of the average signal intensity for each clustered interaction separated by antibody isotype/subclass and cohort. The dotted line represents the cut-off based on the no-DNA control spots. Proteins with signal intensity below the cut-off were set to zero to decrease background noise. Identities of proteins within clusters are provided in Tables S1 and S2 and Figure S5.

All the 215 proteins printed on the array were subjected to cluster analysis to identify proteins with similar reactivity profiles. Two different methods of unsupervised clustering were applied: partitional and hierarchical clustering. Considering all of the possible combinations of antibody reactivity patterns, we used classical multidimensional scaling (MDS) cluster analysis to generate clusters of proteins. In this analysis, the reactivity of each protein was described with the average SI for each cohort. Proteins with an average SI below the cut-off in the evaluated group were considered to be zero and only proteins with an average signal intensity above the cut-off for at least one isotype/subclass were considered for clustering. For partitional clustering, working with 4 antibody isotypes/subclasses and 5 cohorts, proteins fell into one of 7 clusters determined by K-means methodology [40]. To facilitate visualization of the process (and avoid superimposing data points in a 2-dimensional format), we compressed the 20 dimensions into just 2 dimensions (Figure 6A). For hierarchical clustering, a dendrogram was designed using complete linkage [41] to combine two proteins and color coded to match the k-means clusters; identities of the proteins within each cluster can be found in Table S2 and Figure S5. There was good correlation between partitional and hierarchical clustering, indicating that the division of proteins in these groups was robust. To further enhance the visualization process, clustered proteins were distributed in 2 dimensions based on isotype/subclass specific responses of each cohort to each individual protein (Figure 6B).

A number of clusters of interest for vaccine development were observed. Clusters 4 and 5 are characterized by proteins that are moderate to strong targets of IgE and IgG3 or IgG1 responses, respectively, particularly in the CI-Mod and CI-Heavy groups. Cluster 7 predominantly consists of non-reactive proteins and a small handful of proteins that were exclusively targeted by IgG1 responses of the individual sera in the PR group but not the CI or non-endemic control groups. Of the strongly reactive PR IgG1 proteins, *Sm*29 was also recognized by all IgG subclasses as well as IgE and belonged to cluster 2; *Sm*-TSP-2 and *Sm*-CAM-3 on the other hand were uniquely targeted by PR IgG1 and not other isotypes or subclasses and belonged to cluster 7 (Figure 6, Figure S6 and Table S2). Other cluster 7 proteins that were uniquely recognized by PR IgG1 responses, albeit relatively weak responses, included Smp_124240 (Na/K transporting ATPase beta subunit) and Sj_AY915291 (fatty acid CoA synthetase). Both of these proteins have multiple predicted membrane spanning domains (not shown).

### Immune responses to current schistosome vaccine antigens

**Figure 7:**
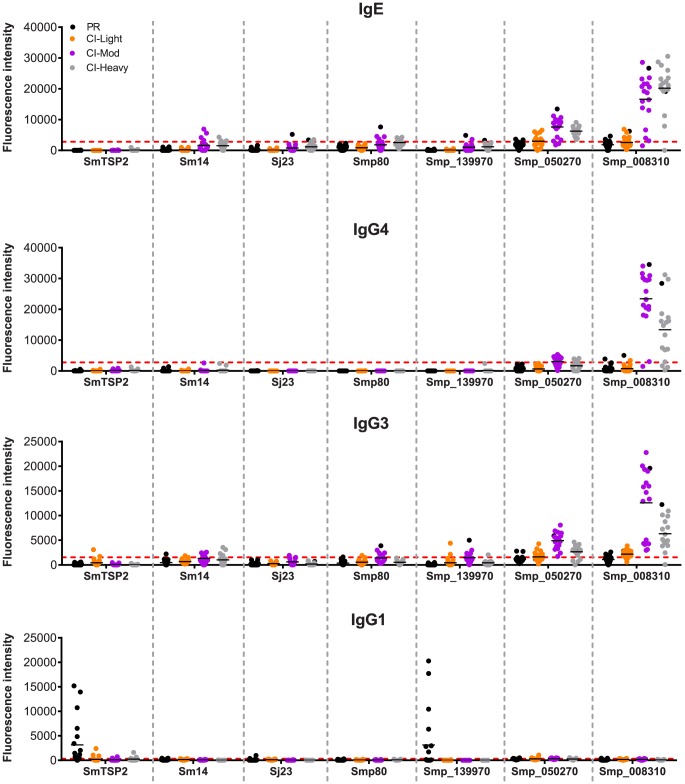
Immunoreactivity of current schistosomiasis vaccine antigens printed on the microarray. Signal intensity values depicting isotype/subclass-specific antibody responses of individual subjects in each cohort to proteins corresponding to known schistosomiasis vaccine antigens and other select RTS proteins that were spotted on the microarray - *Sm-*TSP-2, *Sm-*14, *Sj*-23 and *Sm*p80. Three new RTS antigens from *S. mansoni* were also included: Smp_139970, Smp_050270 and Smp_008310. Only groups from the schistosomiasis endemic area are represented: Putative Resistant (PR - blue), *S. mansoni* chronically infected with low (CI-Light – orange), moderate (CI-Mod - purple) and high (CI-Heavy - gray) intensity infections. The red line is the cut-off calculated as one standard deviation of the no-DNA control spots printed on the array and probed for each specific antibody isotype/subclass.

We examined the antibody recognition profiles of individuals within the cohorts to some of the current antigens that are under various stages of pre-clinical development as human schistosomiasis vaccines, including *Sm*-TSP-2, *Sm*p80 (calpain) and *Sm*14, and bovine vaccines to interrupt zoonotic transmission (*Sj*23). We compared the responses of these known vaccine antigens with selected RTS proteins including the PR IgG1-specific target Smp_139970 (*Sm*-CAM-3) and 2 proteins that were significant targets of IgE and/or IgG4 in CI-Mod and CI-Heavy cohorts, Smp_050270 and Smp_008310 (Figure 7). Different antigens displayed distinct IgE and IgG subclass profiles. *Sm*-TSP-2 was the target of a strong IgG1 response that was unique to the PR group, in agreement with the published literature [26]. Smp_139970 (*Sm*-CAM-3) showed the same recognition profile as that targeting *Sm*-TSP-2. Mean SI values for IgE were below the cut-offs for all of the vaccine antigens, however varying numbers of individuals in the CI-Moderate and CI-Heavy groups were positive for some of the antigens, although SI values were weak compared with other RTS proteins such as Smp_008310. Similarly, IgG4 responses were mostly below the cut-off for the established vaccine antigens. The mean IgG3 responses were mostly negative but weakly positive for Smp80 and *Sm*-CAM-3 in the CI-Mod cohort. IgG1 responses to the known (and potentially new) vaccine antigens were the most noteworthy in terms of unique recognition by the PR cohort: strong IgG1 responses to both *Sm*-TSP-2 and *Sm*-CAM-3 were detected in just the PR group and none of the CI groups (Figure 7).

## Discussion

Herein we describe the first immunomics-based approach to study the humoral immune response to a multi-cellular pathogen. The “immunome” can be defined as the entire set of antigens or epitopes that interface with the host immune system [42]. Recent advances in high order multiplexing, or megaplexing, such as the protein microarray discussed below, provide a practical, high-throughput and affordable approach to estimating the immunomic profiles of humans or animals to a pathogen [43], [44]. This approach permits investigators to assess the repertoire of antibodies created in response to infections or vaccinations from large collections of individual sera. Further, it can be used to perform large-scale sero-epidemiological, longitudinal and sero-surveillance analyses not possible with other technologies.

Numerous passive transfer studies [45], [46] support the critical role of antibodies in immunity to *S. mansoni* infection in rodent models. Perhaps the most compelling evidence that the humoral immune response targets the tegument and can kill parasites comes from studies with rats, which are semi-permissive to *S. mansoni* [47], [48]. Resistance to schistosomiasis can be passively transferred via serum from resistant rats, and protective antibodies can be removed by adsorption on the surface of schistosomes [49]. Indeed, two of the recombinant antigens used on our array - *Sm*-TSP-2 and *Sm*29 - have proven efficacious in a mouse challenge model and were the major targets of single chain antibodies from resistant rats adsorbed from the surface of live schistosomes [50].

The role of antibodies in protective immunity against schistosomiasis in humans is, however, somewhat contentious. Unlike experimentally infected rats, protective immunity to schistosomes in humans develops slowly (over many years) and is rarely sterilizing in nature. Distinct molecular mechanisms are thought be critical in the acquisition of immunity in different transmission scenarios. For example, some individuals can successfully mount a protective antibody-mediated response that targets adult *S. mansoni* antigens after repeated rounds of PZQ therapy [51], [52] – this drug-induced resistance is mediated by IgE and T helper type 2 (Th2) cytokines, and can be accelerated and augmented by repeated drug treatment [11], [53], [54]. We show here that CI individuals make robust IgE responses to many antigens, and the number of antigens recognized increases with increasing intensity of infection as measured by eggs per gram of feces. This would appear to contrast with the protective role that is often associated with IgE in helminth infections, including schistosomiasis [55].

In contrast to drug-induced resistance to schistosomiasis, naturally acquired resistance has been reported in a subset of people who have constant exposure to schistosomes but have never been treated with PZQ (exemplified by the PR cohort in our study) – these individuals generate robust T cell responses against the surface of the larval schistosomulum and are characterized by elevated levels of IFN-γ [35], [56]–[58]. We show here that PR individuals, despite constant exposure to *S. mansoni*, do not appear to mount a strong IgE response to the proteins on the array. Unlike CI individuals, PR subjects are repeatedly negative for schistosome eggs in the feces, and are therefore unlikely to receive the IgE-inducing stimulus of eggs trapped in the bowel wall and the subsequent hepato-portal inflammation that typifies chronic schistosomiasis. PR individuals are likely to kill juvenile schistosomes before they reach sexual maturity, either in the skin or the lungs. The strong recognition of just a handful of tegument antigens by IgG1 from PR individuals therefore implies a protective role for IgG antibodies (and/or T cells) targeting these proteins.

A major outcome of this study is the development of a tool by which the immunogenicity and probable safety profile (i.e. IgE recognition) of an antigen can be rapidly assessed, and a putative association of that antigen-antibody interaction with resistance or susceptibility to infection inferred. In terms of vaccine antigen discovery, we employed the following principles to a given antigen: (1) up-selection for further evaluation based on preferential recognition by IgG1 and/or IgG3 from the PR but not the CI cohorts; (2) down-selection based on recognition by IgE from either PR or CI individuals. Our rationale for down-selection of IgE-inducing antigens is primarily due to safety concerns [19]. In a recent phase I clinical trial of the of the *Na*-ASP-2 hookworm vaccine in a *Necator americanus*-endemic area of Brazil, hookworm exposed individuals were vaccinated with a recombinant protein which, despite proving safe and immunogenic in helminth-naïve individuals in the USA [59], induced an immediate hypersensitivity (urticarial) response in vaccinees, resulting in suspension of the clinical trial and further development of this antigen as a vaccine [39]. The allergenicity of the hookworm vaccine was linked to pre-existing IgE to parasite-derived *Na-*ASP-2 in the circulation of exposed individuals. Rather than completely excluding antigens that are the targets of pre-existing IgE responses from further development as vaccine antigens, we suggest that IgE antigens that associate with resistance might be carefully progressed towards studies that assess their anaphylactogenic potential, particularly given the large body of data that shows a protective role for IgE in resistance to human helminths. For a vaccine targeting infants prior to natural exposure to schistosome-infected water, a vaccine that incorporates antigens which are the targets of IgE in older individuals is feasible, and might even harness the putative protective capacity of this immunoglobulin isotype as children first become exposed to the parasite and undergo boosting and isotype class switching.

Using these criteria described above to set the parameters for multi-dimensional clustering, a small number of antigens that made up cluster 7 are noteworthy as targets of IgG1 from the PR cohort but not IgE from any cohort. One of these antigens, *Sm*-TSP-2, was already known to be a selective target of PR IgG responses [26], and its recognition profile on the microarrays served to confirm its potential as a vaccine antigen, as well as the utility of our approach for identifying protective antigens. At least three RTS proteins from cluster 7 were noteworthy as targets of PR IgG1 but not IgE from any exposed cohort. Of these antigens, the strongest IgG1 response was aimed at Smp_139970 (*Sm*-CAM-3). *Sm-cam-3* mRNA (and its *S. japonicum* ortholog, contig 8758) is upregulated in cercariae [60], [61] (Figure S4A) and encodes a member of the calmodulin family of calcium-sensing proteins. Calmodulins respond to changes in calcium ion concentrations by undergoing a conformational change upon binding, which in turn, facilitates interactions with other signaling proteins. Using gene silencing and quantitative parasite motility assays, a schistosome calmodulin dependant kinase (CamKII) was shown to minimise the impact of PZQ treatment against adult *S. japonicum* [62]. *Sm*-CAM-3 is a small (∼8 kDa) protein that contains a single Ca^2+^ binding EF-hand motif and shares moderate homology with mammalian and other parasite calmodulins, the majority of which are larger than *Sm*-CAM-3 and contain multiple EF-hands. Of the four calmodulin family members present in *S. mansoni*, two have been characterized (*Sm*CaM1 and *Sm*CaM2) and were detected in the tegument of adult worms [30], [63] and the epidermal and tegumental layers of larval stages in the snail host [64]. Indeed RNAi-mediated silencing of these genes resulted in stunted larval development [64]. Generic calmodulin antagonists have been shown to inhibit the *in vitro* growth and egg-hatching ability of schistosomes [64], [65] and the growth of *Plasmodium* [66] but have limited use as anti-parasitic interventions due to the highly conserved nature of calmodulins across species. It is therefore noteworthy that *Sm-*CAM-3 shares only ∼20% identity with primate calmodulins (Figure S4B), supporting its development as a safe schistosomiasis vaccine that is unlikely to induce antibodies which cross-react with homologous human proteins. Although *Sm*-CAM-3 is immunogenic in PR individuals, it is a small protein and might not be overly immunogenic as a subunit vaccine. An ideal schistosomiasis vaccine might therefore incorporate *Sm*-CAM-3 as part of a larger chimeric construct with other vaccine antigens such as Smp80-calpain or *Sm*-TSP-2, a strategy that was recently shown to boost efficacy in a mouse challenge model [67]. Two other cluster 7 RTS proteins were unique targets of just IgG1 in the PR cohort, albeit with relatively weak responses – Smp_124240 (Na/K transporting ATPase beta subunit) and Sj_AY915291 (fatty acid CoA synthetase). Both proteins have multiple predicted membrane spanning domains, and warrant expression of defined extracellular domains in a cell-based system for further investigation as vaccine antigens.

In addition to *Sm*-TSP-2, two other high profile vaccine antigens, Smp80-calpain (Smp_137410) and Sm14 (Smp_095360), were contained within cluster 7. Although some individuals mounted IgG and to a lesser extent IgE responses to these RTS products, the mean SI values for any isotype/subclass to either antigen were below the cut-offs. The apparent absence of a positive mean SI (indicative of a strong antibody response) to an RTS protein on the array should be treated with due caution – the benefit of RTS protein production is its inherent high-throughput nature and its suitability for printing onto arrays in nanoliter quantities. One of the major limitations of RTS protein production however is the absence of complex secretory machinery and the dependence on secretory pathways for correct folding and processing of some proteins. While there are RTS systems that contain eukaryotic microsomal membranes, these systems are not widely used for protein microarray production, and would add substantial cost to the production of large arrays. *Sm*-TSP-2 was available to us in recombinant, cell-derived form, and was not successfully translated in RTS form. Smp80-calpain and Sm14 were successfully produced in RTS form (Figure S1), but we cannot guarantee faithful replication of all the native epitopes. We therefore urge caution in the interpretation of a “negative” result using this microarray approach, but we have confidence in assigning a “positive” antibody response.

Proteome microarrays have been used to identify candidate vaccine antigens for a number of infectious diseases of viral and bacterial origin [43]. To date, *Plasmodium* is the only eukaryotic parasite for which proteome microarrays have been described [68]. Screening of *P. falciparum* proteome arrays with sera from well-defined clinical cohorts resident in malaria-endemic areas [68]–[70] and recipients of radiation attenuated vaccines [71] has resulted in the identification of a suite of new vaccine antigens, some of which have proven efficacious in mouse models of malaria (DLD, unpublished observations).

Our proteome microarray included a small number of carefully selected *S. mansoni* and *S. japonicum* proteins, working on the assumption that tegument surface proteins are most likely to be the targets of PR protective immune responses. Based on our findings here, notably the large number of immunogenic proteins, a second generation array that consists of a much larger number of *S. mansoni* proteins, both extracellular and intracellular, would likely yield many more immunogenic proteins, including potential vaccine and diagnostic antigens. This is particularly relevant in the context of antigen discovery using sera from individuals who have developed resistance to schistosomiasis after repeated rounds of PZQ therapy (drug induced resistance - DIR), where the immune response is primarily aimed at intracellular molecules released by dying worms [72] or other means of protein export such as exosomes [73]. We are now screening our array (and subsequent generation arrays) with sera from DIR individuals. Indeed, if a schistosomiasis vaccine is developed, it is likely to be incorporated into an integrated control program that couples chemotherapy with vaccination [19], so a comprehensive assessment of the targets of DIR immunity will prove to be an essential component of future schistosomiasis vaccine antigen discovery.

We have shown here that proteome microarrays provide an ideal means by which to explore humoral immunity and vaccine antigen discovery for parasitic helminth infections. The approach is less labor intensive and more sensitive than traditional immunoproteomics based approaches that employ 2D Western blots followed by protein extraction from SDS gels [72]. Moreover, antigens can be readily up-selected for their protective properties and down-selected for potentially deleterious allergic properties, in a high-throughput fashion with large numbers of sera. The power of this technology lies with the nature of the assembled cohorts – whether they are well-characterized groups of naturally resistant and susceptible individuals, or animals that have been experimentally rendered resistant by vaccination (e.g. irradiated schistosome cercariae [74], [75]). With the recent sequencing of the *S. haematobium* genome [27], and the enormous burden of disease that is attributed to urogenital schistosomiasis in Sub-Saharan Africa [76], it is now essential to apply a systems vaccinology approach to the integrated control of all the major schistosomes infecting humans. Future efforts will explore the replication of conformational epitopes in prokaryotic (as done herein) versus eukaryotic (eg. insect cell lysates) RTS systems, and larger protein microarrays containing the entire parasite secretome will be produced to allow a more comprehensive screen and ensure that a pipeline of schistosomiasis vaccine antigens is generated for progression towards clinical trials.

## Materials and Methods

### Ethics statement

All subjects provided written informed consent using forms approved by the Ethics Committee of Centro de Pesquisa René Rachou (reference number Fev/04), the Federal Institutional Review Board of Brazil or CONEP (25000.029.297/2004-58) and the George Washington University School of Medicine Institutional Review Board (GWUMC IRB# 100310).

### Study cohorts

Individuals aged 18–60 (inclusive) from a *S. mansoni* endemic areas in Minas Gerais State, Brazil were followed longitudinally. Individuals were determined to be Putative Resistant (PR) if they had regular contact with infected water as determined by water contact studies and surveys for infected snails [26], and no *S. mansoni* eggs in their feces after three days of examination of fecal samples by ether sedimentation and Kato Katz fecal thick smears (2 slides per fecal sample) over a period of 10 years of investigation (n = 20). The PR group were matched with sera taken from individuals deemed to be chronically infected (CI) with *S. mansoni* as determined by the fecal exam methods described above and stratified in the following groups by the intensity of *S. mansoni* infection as expressed in eggs per gram of feces (epg) by Kato Katz fecal thick smear: CI-Light (n = 30; epg <100), CI-Moderate (n = 20; epg = 101–400) and CI-Heavy (n = 20; epg>401). Individuals in each CI intensity strata were age, sex, and water contact matched with a PR individual. The PR and some of the CI sera were the same as those used in Tran *et al*. [26]. We included negative control groups from non-endemic areas of both Brazil (Belo Horizonte, Minas Gerais; n = 12) and the U.S. (n = 12). Donor sera from the U.S. were taken at the George Washington University under an IRB approved protocol. Table S6 contains the demographic information on the different cohorts utilized in this study.

### Recombinant protein expression and printing

A subset of potentially immunogenic open reading frames (ORFs) were selected for expression and printed from publically available coding sequences for *S. mansoni* (n = 63) and *S. japonicum* (n = 214) [38]. Most of these sequences were selected based on bioinformatic, proteomic and transcriptomic data using the following criteria: high sequence homology among the two schistosome species; expression in the immunologically vulnerable schistosomulum stage; predicted or known to be localized on or in the parasite tegument; and limited sequence similarity with mammalian homologs. Primer design and PCR amplification from *S. mansoni* and *S. japonicum* cDNA libraries were performed as described [38]. Amplicons were cloned into the custom made pXi T7 vector containing N-terminal 10-His and C-terminal HA tags by homologous recombination, as described previously [77]. Of the sequences selected, 88% (n = 244) were successfully amplified and the resultant plasmids purified, and the inserts were verified by PCR and sequencing [38] (Table S1). A total of 217 high-yielding plasmids (45 from *S. mansoni* and 172 from *S. japonicum*) with correct inserts were expressed in an *in vitro* cell-free system based on *Escherichia coli* ribosomes (Roche RTS 100), and the protein extracts were contact-printed without purification onto nitrocellulose glass ONCYTE slides. As controls, the following purified recombinant antigens expressed in yeast or *E. coli* were printed onto the array in two dilutions (0.1 and 0.3 mg/ml): *Sm-*TSP-2 (Smp_181530), *Sm*29 (Smp_072190), *Sj*-TSP-2/238 (NCBI ABQ44513) and *Sj* silencer (NCBI AAP06461). Non-schistosome control proteins/RTS reactions were also spotted onto the microarray as described [38], and included Epstein Barr virus protein EBNA-1 as well as purified human immunoglobulins. The printed *in vitro* expressed proteins were quality checked using antibodies against incorporated N-terminal poly-histidine (His) and C-terminal hemagluttinin (HA) tags. The efficiency for *in vitro* expression was higher than 95%, where positive features were considered to have detectable His or HA tags (Figure S1).

### Probing of protein microarrays with human sera

Sera were pre-adsorbed for anti-*E. coli* antibodies by rocking for 30 min at RT with *E. coli* lysate before probing of arrays. Protein arrays were blocked in blocking solution (Maine Manufacturing) for 2 hours at RT prior to probing with human sera (diluted 1∶50) at 4°C overnight with gentle constant rocking [71]. Arrays were washed 3 times for 5 min with TBS/0.05% Tween 20 (TTBS) then isotype and subclass specific responses were detected using biotinylated monoclonal antibodies against human IgG1, IgG3, IgG4 (Sigma) and IgE (Hybridoma Reagent Laboratory, Baltimore, MD) diluted 1∶100 for 2 h at RT. Arrays were washed again then incubated for 2 h in streptavidin Cy-5 diluted 1∶400 and washed with TTBS followed by TBS then MQ water, 3×5 min in each solution. Air-dried slides were scanned on a Genepix 4200AL scanner (Molecular Devices) and signal intensities (SI) quantified using the ScanArray Express Microarray Analysis System Version 3.0 (Molecular Devices). Raw SI were corrected for spot-specific background using the Axon GenePix Pro 7 software. Data were analyzed using the “group average” method [78] whereby the mean SI was considered for analysis. Briefly, the SI for negative control spots (empty vector) was calculated for each serum and each antibody type. This value was considered as the background and was subtracted from the SI of each protein spot. To determine if an antigen was recognized a cut-off for each antibody type was defined. The cut offs were defined as one standard deviation above the average of the negative control spots for all groups of sera (Negative BZL, Negative USA, PR, CI-Light, CI-Mod, CI-Heavy) after correcting for spot-specific background. The cut offs were- IgE: 2828.8; IgG4: 2767.3; IgG3: 1557; and IgG1: 316.4.

### Spatial projection and clustering analysis

All 217 RTS proteins as well as purified *E. coli*-derived *Sm*29 and yeast-derived *Sm*-TSP-2 were used to conduct a spatial analysis. When mean SI was below the cut-off for a given antibody isotype/subclass, the SI value was adjusted to zero for this analysis only. To identify clusters containing proteins with the same antibody reactivity profiles we generated a distance matrix estimated from the pairwise Euclidian distance of log transformed SI for each antigen based on the cut-off values for each antibody isotype/subclass in the different cohorts. Complete linkage clustering methodology was used to create a dendrogram analysis of pairwise Euclidian distances for each protein according to the equation below:
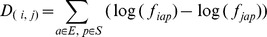



Where *D_(i,j)_* represents the distance between the proteins *i* and *j*, *E* is the antibody isotypes (IgG1, IgG3, IgG4 and IgE) and *S* is the set of individual subjects; *f _iap_* is the fluorescence signal relative to antibody isotype present in the serum of subject *p* reactive against protein *i*; *f _jpa_* is the fluorescence signal for the same sample against protein *j*. To provide a visual representation of each distance matrix, we used a multidimensional scaling (MDS) plot with two dimensions (2D). The unsupervised methodology k-means algorithm with 1,000 interactions [40] was used to define seven clusters. Clusters were validated using clValid, a R software package for cluster validation [79]. The distance matrix, MDS, clustering and graphing were performed using the R software platform (www.r-project.org) [80]. Graphics representing specific relativities that characterized each cluster were designed using GraphPad Prism 5.0. The raw SI values grouped into clusters are provided in Table S2.

### Statistical analyses

Kruskal-Wallis with Dunn's multiple comparison test was used to compare more than two independent samples (Figure 1) and to calculate the statistical differences between the groups if the protein was classified as reactive (Figures 2–5). Correlations were calculated using the Spearman Test. Statistical analyses were performed with GraphPad Prism 5.0.

## Supporting Information

Figure S1 **Quality control probing of the schistosome protein array with anti-6His and anti-HA antibodies.** Control proteins on the array are denoted by colored boxes and the corresponding key at the bottom of the image. *Schistosoma* proteins correspond to spots present inside the orange dashed lines. Other proteins on the array that are not contained within boxes are hookworm proteins that are not considered within the context of this study.
(EPS)

Figure S2 **Consistent IgG1 immunoreactivity of all cohorts to the Epstein Barr virus EBNA-1 protein.** IgG1 response was evaluated in all subjects to EBNA-1 protein spotted at a concentration of 0.1 mg/ml (upper panel) and 0.3 mg/ml (lower panel) on the protein array. Negative BZL control (Neg-BZL), Putative Resistant (PR), chronically infected with *S. mansoni* at low (CI-Light), moderate (CI-Mod) and high (CI-Heavy) infection intensities. Red line is the cut-off calculated by the average of the signal intensity plus one standard deviation of the no-DNA control spots present on the array.
(EPS)

Figure S3 **Anti-soluble worm antigen preparation (SWAP) IgG subclass responses.** ELISA was performed by coating microtiter plates with SWAP and probing with sera from the different cohorts followed by subclass specific secondary antibodies. Negative Brazilian controls from a non-endemic area for schistosomiasis (BRZ-Neg), Putative Resistant (PR), chronically infected with *S. mansoni* at low (CI-Light), moderate (CI-Mod) and high (CI-Heavy) infection intensities.
(TIF)

Figure S4 **Developmental expression of** ***Sm-cam-3*** **(Smp-139970) and its** ***S. japonicum*** **ortholog (contig8758) during the schistosome lifecycle, as obtained from public databases.** (A) Left: developmental expression of *S. japonicum* contig8758 [60]; Middle: developmental expression of *S. mansoni* TC16561 ( = Smp-139970) during the cercaria to schistosomulum transformation determined by microarray analysis [61]; Right: developmental expression of Smp_139970 in *S. mansoni* during the cercaria to lung-stage schistosomulum transformation determined by RNA Seq reads [81]. Mira: miracidia, Sporo: sporocysts, Cerc: cercariae, Lung: 3 day lung schistosomula, M4-6-7: adult males from mice at 4-6-7 weeks post cercarial challenge, F4-6-7: adult females from mice at 4-6-7 weeks post cercarial challenge, 3-5-24 hr: 3-5-24 hr schistosomula post mechanical transformation of cercariae, Adults: mixed male and female adults from mice. (B) Multiple sequence alignment of *Sm*-CAM-3 with its closest homologues from *S. mansoni* (*Sm*-CAM-4; NCBI XP_002574739) and non-human primates (*Macaca mulatta* calmodulin; NCBI EHH18861).
(TIF)

Figure S5 **Correlations between IgG4 and IgE responses in each different cohort of schistosome exposed individuals.** Dots represent the mean signal intensity per reactive protein within each group. Correlations were performed using linear regression (color coded solid lines and r2 values as indicated) and 95% confidence intervals are denoted by dashed lines. Groups are putative resistant (PR), *S. mansoni* chronically infected with low (CI-low), moderate (CI-Mod) and heavy (CI-Heavy) intensity infections.
(TIF)

Figure S6 **Dendrogram showing the multi-dimensional clustering of immunoreactive proteins.** Multi-dimensional clustered distribution of all proteins according to the antibody isotype/subclass responses in distinct cohorts. Proteins formed 7 clusters, defined by the following colors: cluster 1 - black (4 proteins); cluster 2 – blue (11 proteins); cluster 3 – grey (5 proteins); cluster 4 – green (31 proteins); cluster 5 – magenta (47 proteins); cluster 6 – orange (11 proteins) and cluster 7 – red (106 proteins).
(TIF)

Table S1 **List of antigens printed with annotation and protein sequences.**
(XLSX)

Table S2 **List of immunoreactive proteins divided and color-coded by clusters according to the multi-dimensional cluster analysis (Figure S5).**
(XLSX)

Table S3 **List of immunoreactive proteins by isotype.**
(XLSX)

Table S4 **Statistical analyses for all immunoreactive proteins divided by isotype/subclass.**
(XLSX)

Table S5 **Spearman correlations between isotype/subclass specific antibody responses.**
(XLSX)

Table S6 **Demographic characteristics of the study groups.**
(DOCX)
