# Supplementary figures and images for: An Immunomics Approach to Schistosome Antigen Discovery: Antibody Signatures of Naturally Resistant and Chronically Infected Individuals from Endemic Areas
Source: PLoS Pathog. 2014 Mar 27;10(3):e1004033. doi: 10.1371/journal.ppat.1004033 (PMC3968167; doi:10.1371/journal.ppat.1004033)

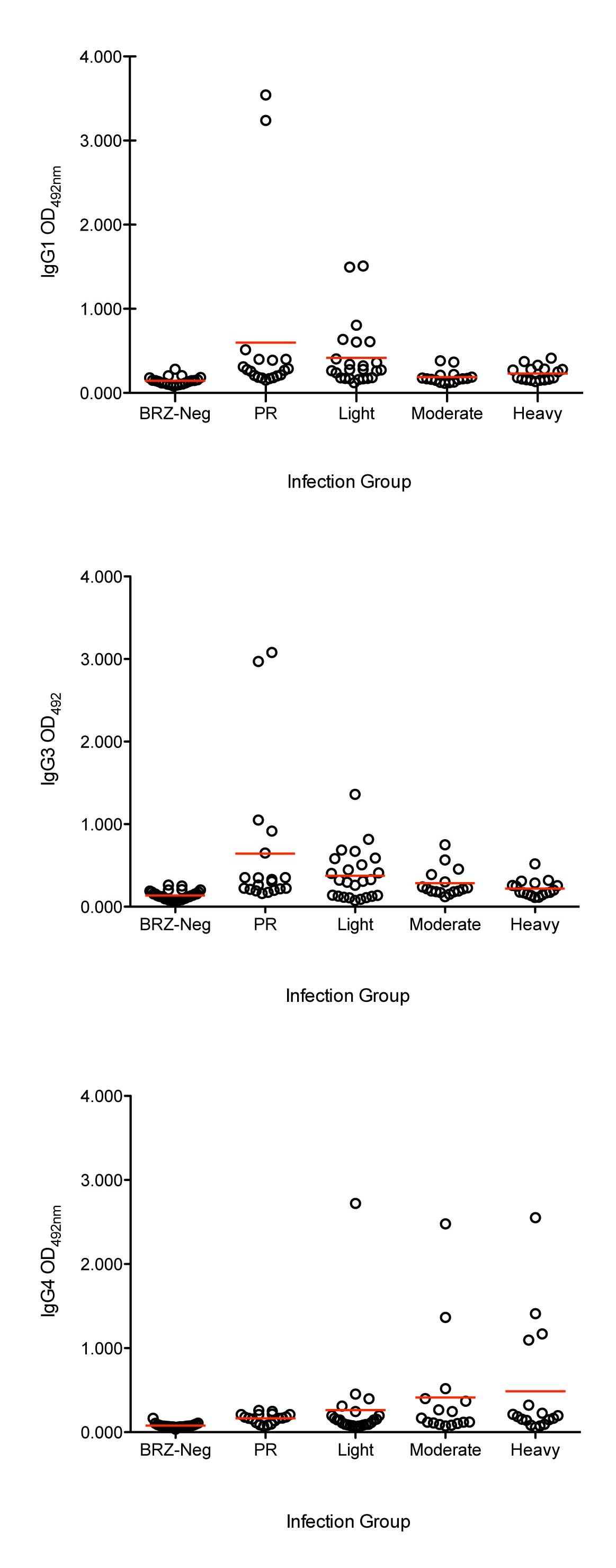

Supplement: Figure S3 — Anti-soluble worm antigen preparation (SWAP) IgG subclass responses. ELISA was performed by coating microtiter plates with SWAP and probing with sera from the different cohorts followed by subclass specific secondary antibodies. Negative Brazilian controls from a non-endemic area for schistosomiasis (BRZ-Neg), Putative Resistant (PR), chronically infected with S. mansoni at low (CI-Light), moderate (CI-Mod) and high (CI-Heavy) infection intensities. (TIF) [file ppat.1004033.s003.tif]

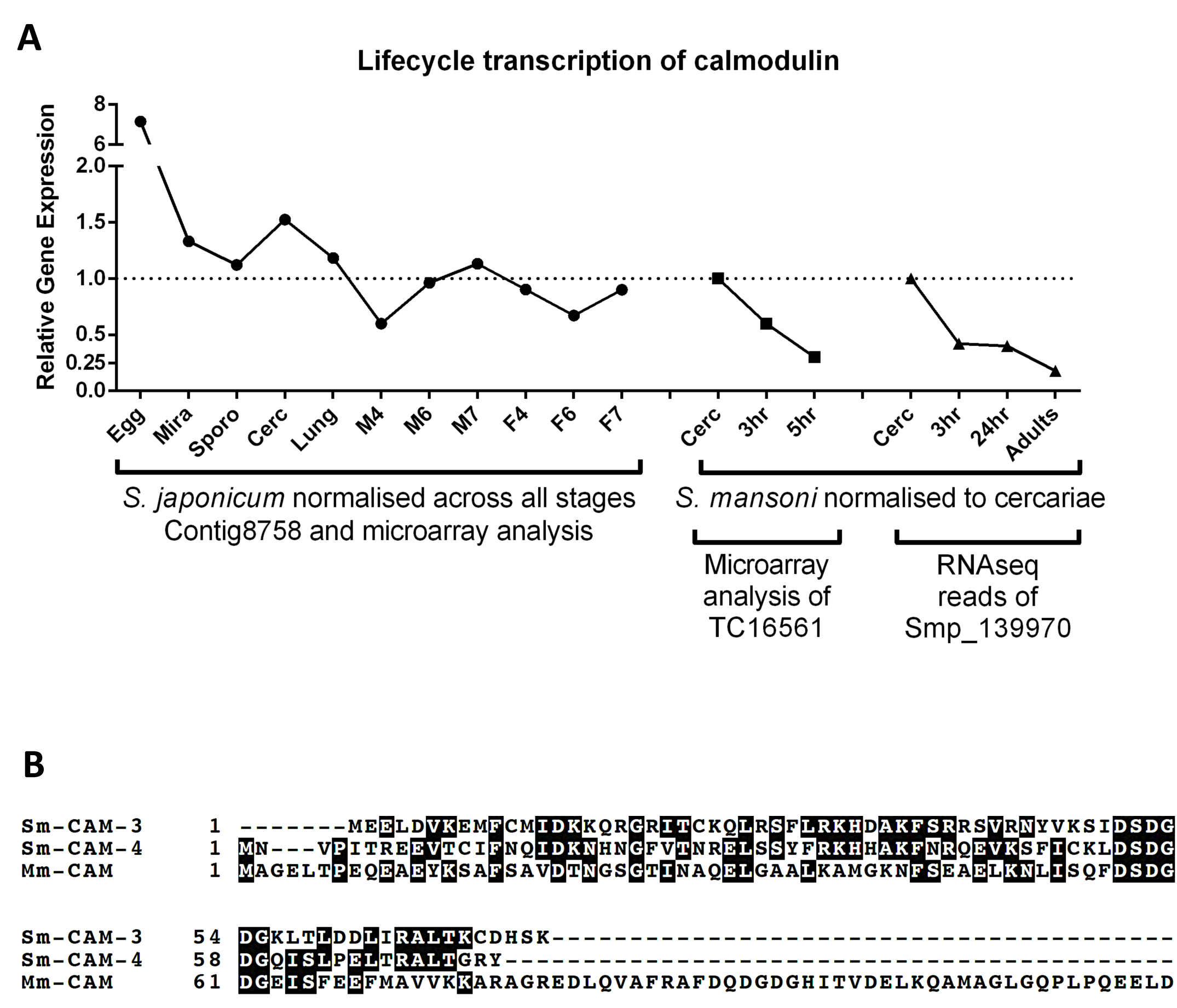

Supplement: Figure S4 — Developmental expression of Sm-cam-3 (Smp-139970) and its S. japonicum ortholog (contig8758) during the schistosome lifecycle, as obtained from public databases. (A) Left: developmental expression of S. japonicum contig8758 [60]; Middle: developmental expression of S. mansoni TC16561 ( = Smp-139970) during the cercaria to schistosomulum transformation determined by microarray analysis [61]; Right: developmental expression of Smp_139970 in S. mansoni during the cercaria to lung-stage schistosomulum transformation determined by RNA Seq reads [81]. Mira: miracidia, Sporo: sporocysts, Cerc: cercariae, Lung: 3 day lung schistosomula, M4-6-7: adult males from mice at 4-6-7 weeks post cercarial challenge, F4-6-7: adult females from mice at 4-6-7 weeks post cercarial challenge, 3-5-24 hr: 3-5-24 hr schistosomula post mechanical transformation of cercariae, Adults: mixed male and female adults from mice. (B) Multiple sequence alignment of Sm-CAM-3 with its closest homologues from S. mansoni (Sm-CAM-4; NCBI XP_002574739) and non-human primates (Macaca mulatta calmodulin; NCBI EHH18861). (TIF) [file ppat.1004033.s004.tif]

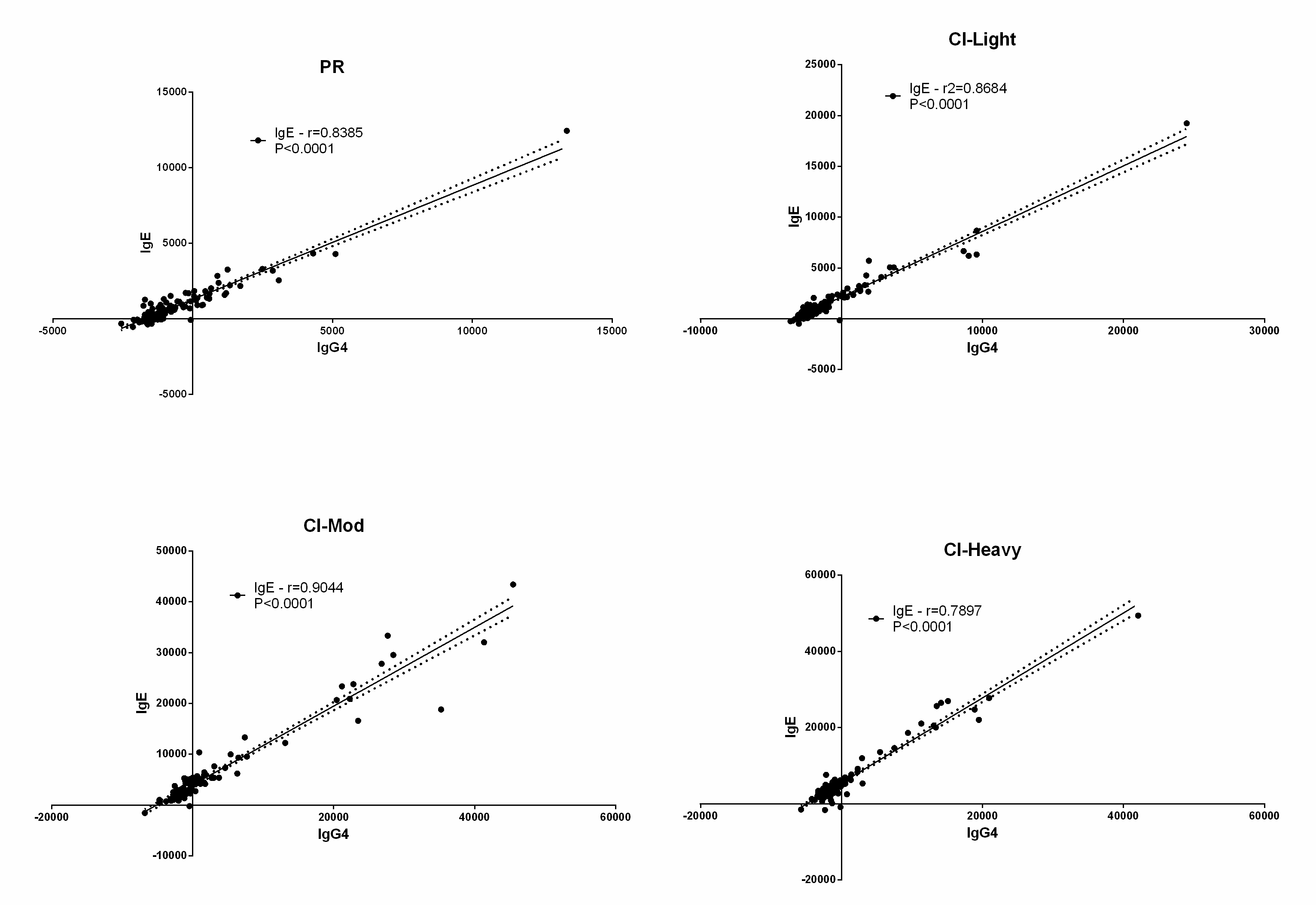

Supplement: Figure S5 — Correlations between IgG4 and IgE responses in each different cohort of schistosome exposed individuals. Dots represent the mean signal intensity per reactive protein within each group. Correlations were performed using linear regression (color coded solid lines and r2 values as indicated) and 95% confidence intervals are denoted by dashed lines. Groups are putative resistant (PR), S. mansoni chronically infected with low (CI-low), moderate (CI-Mod) and heavy (CI-Heavy) intensity infections. (TIF) [file ppat.1004033.s005.tif]

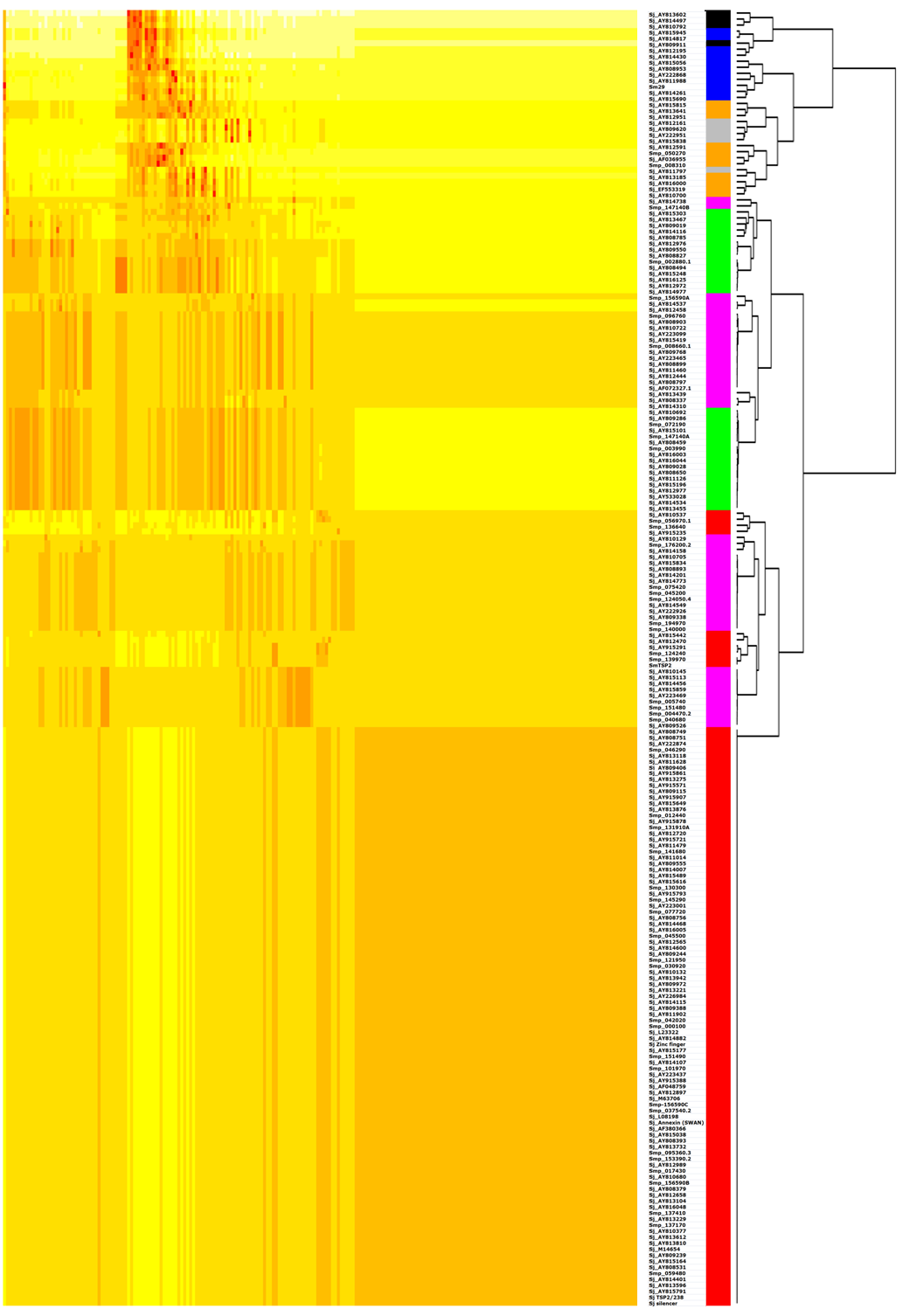

Supplement: Figure S6 — Dendrogram showing the multi-dimensional clustering of immunoreactive proteins. Multi-dimensional clustered distribution of all proteins according to the antibody isotype/subclass responses in distinct cohorts. Proteins formed 7 clusters, defined by the following colors: cluster 1 - black (4 proteins); cluster 2 – blue (11 proteins); cluster 3 – grey (5 proteins); cluster 4 – green (31 proteins); cluster 5 – magenta (47 proteins); cluster 6 – orange (11 proteins) and cluster 7 – red (106 proteins). (TIF) [file ppat.1004033.s006.tif]
