# Supplementary material for: An Immunomics Approach to Schistosome Antigen Discovery: Antibody Signatures of Naturally Resistant and Chronically Infected Individuals from Endemic Areas
Source: PLoS Pathog. 2014 Mar 27;10(3):e1004033. doi: 10.1371/journal.ppat.1004033 (PMC3968167; doi:10.1371/journal.ppat.1004033)
Supplement: Table S6 — Demographic characteristics of the study groups. (DOCX) [file ppat.1004033.s012.docx]

| Table S6. Demographic characteristics of the study groups | | | | | | |
| --- | --- | --- | --- | --- | --- | --- |
|  | |  | Age | | Sex | |
| Group | | N | Mean | 95%CI | M | F |
|  | |  |  |  |  |  |
| Control groups | |  |  |  |  |  |
| Brazil Negative | | 12 | 28.17 | (24.27, 32.06) | 5 | 7 |
| Non-endemic Negatives | | 10 | 23.20 | (20.09, 26.31) | 12 | 0 |
|  | | |  |  |  |  |
| *S. mansoni* infection groups* | | |  |  |  |  |
| Putative Resistant | 19 | | 35.63 | (24.44, 45.82) | 12 | 7 |
| Light infection | 24 | | 22.17 | (14.33, 30.00) | 11 | 13 |
| Moderate Infection | 16 | | 31.69 | (24.18, 39.19) | 4 | 12 |
| Heavy Infection | 17 | | 28.35 | (23.68, 33,00) | 9 | 8 |
| * Inclusion criteria required permanent residence in the *S. mansoni* endemic area and no previous treatment with praziquantel. | | | | | | |
